# Non-Fire Related Carbon Monoxide Poisoning in Sichuan, China: A 9-Year Study (2008–2016)

**Published:** 2019-03

**Authors:** Fan CHEN, Yi YE, Qingtao WEI, Jianxia CHEN, Hao WU, Youyi YAN, Linchuan LIAO

**Affiliations:** Department of Forensic Analytical Toxicology, West China School of Basic Science and Forensic Medicine, Sichuan University, Chengdu, China

**Keywords:** Non-fire, Carbon monoxide, Poisoning, Retrospective study

## Abstract

**Background:**

Carbon monoxide (CO) poisoning is one of the most important intoxications in the modern world. This study aimed to identify the characteristics of CO poisoning deaths in Sichuan province in the west of China.

**Methods:**

Data on fatal non-fire-related carbon monoxide poisoning in Sichuan from 2008 to 2016 were obtained from the Department of Forensic Analytical Toxicology of Sichuan University and were analyzed by the month and year of registration of death, sex, age group, manner of death, source of CO, and location of CO exposure. Comparing with the previous studies carried out in Wuhan and Shanghai to identify the regional differences of CO poisoning in China.

**Results:**

A total of 165 non-fire related CO poisoning cases including 237 victims were recorded. Over 90% of the victims died from accidental poisoning. Non-fire related CO poisoning occurred more frequently in winter months and was most prevalent in individuals aged between 18 and 60 yr old. Showering gas accident and coal or charcoal burning was found to be the major source of CO in accident and in suicide cases, respectively. Furthermore, significant regional differences of CO poisoning have been detected in the manner of death and the source of CO.

**Conclusion:**

These findings will be valuable in the targeted prevention of non-fire related CO poisoning in China.

## Introduction

Carbon monoxide (CO) is a colorless, odorless, tasteless toxic gas produced by incomplete combustion of carbon-based fuel. The affinity of CO for hemoglobin is 200–250 times greater than that of oxygen (1), resulting in tissue hypoxia. CO is still responsible for numerous deaths worldwide (2). The epidemiological studies of CO poisoning had been carried out in many countries and regions, such as United States, Turkey, Greece, and European, etc. (3–7).

Carbon monoxide poisoning mainly resulted from accidents or suicides, and rarely resulted from homicides in forensic practice. Motor vehicle exhaust, fire, charcoal burning in confined spaces, fireplaces and defective or improperly ventilated gas-powered appliances are the common sources of CO (3–8). Death due to CO poisoning is identified by values of carboxyhemoglobin (COHb) saturation above 50% in postmortem blood (9). COHb levels less than 10% usually are not associated with symptoms (10). As a general rule, if two or more individuals are found dead indoors, or any type of enclosure and there is no external evidence of trauma, the most probable cause of death is an asphyxiating gas and almost invariably CO (2).

Recently, the epidemiological studies of CO poisoning in Wuhan and Shanghai were published (11, 12). The two cities are located in the central and the east of China, respectively. Due to the vast geographic expanse of China, regional differences could exist in many aspects, such as energy distribution, natural conditions, and economy, etc. Hence, the aim of the present study was to characterize the CO poisoning deaths in Sichuan (a province in western China). It could be a complementary data of the previous studies. Moreover, a comparison between this study and the previous ones would be made in order to get more comprehensive information about CO poisoning deaths in mainland China.

## Methods

This retrospective study covering data collected from Jan 2008 to Dec 2016 records of Department of Forensic Analytical Toxicology of Sichuan University, in Chengdu, China. Cases where COHb saturation in postmortem blood showed CO intoxication were selected. Fire-related cases were excluded. The study was approved by the Sichuan University Ethical Committee.

Non-fire related CO poisoning deaths were analyzed and classified according to the following epidemiological parameters: the month and the year when the death happened, the age and the gender of the victims, the manner of death, the source of CO, and the location of CO exposure. Detailed information of death was provided by the police. The determination of COHb in postmortem blood samples was performed by double wavelength spectrophotometry (Shimadzu UV-2550).

Chi-squared test and Fisher’s exact test (SPSS 20.0 (Chicago, IL, USA) were used to compare the parameters between different regions. *P*<0.05 was considered to be significant in the comparisons.

## Results

During the period Jan 2008 to Dec 2016, 165 non-fire related CO poisoning cases with 237 deaths were recorded by our department. Yearly and monthly distribution of the non-fire related CO poisoning deaths is showed in Table 1 and 2, respectively. Despite some fluctuation from year to year, there was an increase in the annual number of victims from 2008 to 2016. The highest number of deaths occurred in 2015 with 40 victims, followed by 2012 with 33 victims. The highest non-fire related CO poisoning deaths recorded was in the months of Jan and Dec with 57 victims (24.1%) and 44 victims (18.6%), respectively (Table 2).

**Table 1:** Yearly distribution of the non-fire related CO poisoning (2008–2016)

***Year***	***No. of Victims***	***No. of Cases***
2008	17	13
2009	30	17
2010	19	17
2011	10	7
2012	33	21
2013	28	20
2014	32	22
2015	40	25
2016	28	23
Total	237	165

**Table 2:** Monthly distribution of the non-fire related CO poisoning (2008–2016)

***Month***	***No. of victims***	***No. of cases***
January	57	41
February	34	27
March	27	18
April	17	12
May	5	5
June	14	5
July	5	3
August	6	0
September	3	4
October	7	5
November	18	12
December	44	33

Demographic characteristics of the deaths and information about CO poisoning including the manner of death, the source of CO, and the location of CO exposure are presented in Table 3. Males accounted for 53.6% (n=127) of the non-fire CO-related victims, whereas females represented 46.4% (n=110). The age of the victims ranged from 1 to 68 yr old (mean age, 29.7 yr old), and the age group of 18–60 yr old contained 80.2% of the victims. 87.8% (n=208) of the victims were the result of accidental poisoning, 10.5% (n=25) due to suicide, and the remaining due to homicide (Table 3).

**Table 3:** Comparison of non-fire related CO poisoning among Sichuan, Wuhan and Shanghai

***Variable***	***Sichuan***	***Wuhan ^(11)^***	***P-value***	***Shanghai^(12)^***	***P-value***
Gender (%)					
Male	53.6	51.8	*	52.9	*
Female	46.4%	48.2		47.1	
Age (%)					
<18	16.4	0	*P*<0.001	7.6	*P*<0.01
18–60	80.2	80.4		83.2	
<60	3.4	19.6		9.2	
Manner of death (%)					
Accident	87.8	43.7	*P*<0.001	69.2	*P*<0.01
Suicide	10.5	55.2		30.8	
Homicide	1.7	1.1		0	
Source of CO in suicide (%)					
Coal or charcoal burning	68.0	70.8	*	97.3	*P*<0.001
Gas leak	28.0	29.2		0	
motor vehicle exhaust	4.0	0		0	
CO stored in a bottle/balloon	0	0		2.7	
Source of CO in accident (%)					
Showering gas accident	74.5	28.9	*P*<0.001	62.7	*P*<0.001
Gas leak	16.3	42.1		4.8	
Fuel generator	3.4	0		2.4	
Coal or charcoal burning	3.4	28.9		18.1	
motor vehicle exhaust	2.4	0		9.6	
others	0	0		2.4	
Location of CO exposure (%)					
Hostel/daily rented room	51.5	NA	*	NA	*
Home	43.0	NA		NA	
Vehicle	3.4	NA		NA	
Workplace	2.1	NA		NA	

*: means no statistic difference

NA indicated that the data are not available

Coal or charcoal burning was the main source of CO in suicides, and showering gas accident caused the most accidental poisoning Rental house/hostel and the decedent’s home were the main location of CO exposure.

The comparisons between Sichuan and two other regions (Shanghai and Wuhan) in non-fire related CO poisoning are also shown in Table 3. Sex distribution in these three regions was similar. The frequency of victims under the age of 18 in Sichuan was higher than that in Wuhan (*P*<0.001) and Shanghai (*P*<0.01). There were significant differences between Sichuan and Shanghai in the manner of death and the source of CO. Wuhan showed difference in the manner of death and the source of CO in accident when compared with Sichuan.

## Discussion

CO poisoning deaths can be divided into two types, the fire-related ones, and the non-fire related ones. Only non-fire related CO poisoning deaths were considered in the current study because many others factors (burning, oxygen deficiency, alcohol intoxication, cyanide inhalation and so on) could contribute to the cause of death in fire (13–15). Overall, 165 non-fire related CO poisoning cases resulting in 237 victims were recorded by our department from 2008 to 2016. The evolution of non-fire related CO poisoning deaths showed a fluctuant increase from 2008 to 2016 (Table 1). Although these numbers might not be significant, they could reflect the situation of Sichuan Province as almost all of the cases that suspected CO poisoning in Sichuan would be sent to our department for toxicology analysis. In the present study, non-fire related CO poisonings were more frequent during winter months (Dec, Jan, and Feb), which was consistent with previous studies (3–5, 11, 16, 17). Unintentional CO poisonings were highly correlated with the winter months because the major cause of unintentional poisonings related to domestic heaters and these were likely to be used most heavily in cold (18). In this study, the most common CO source was showering gas accident (about 65% of victims), which may explain the seasonal distribution of CO poisoning.

Males represent a litter higher proportion of the victims (53.6%) than female (Table 3). By contrast, the proportion of male victims in non-fire related CO poisoning was much higher in other countries. For instance, males accounted for 75.6% of the non-fire related CO deaths in England and Wales (16), 75.8%% in western Turkey (4) and 77.0% in United States (3). The reason why male represents a larger proportion of CO poisoning deaths was unclear. One possible reason is that males may have a higher risk of carbon monoxide exposure due to the frequent use of CO emitting equipment or working indoors (19, 20). In this study, only a very few male victims were connected with using of CO emitting equipment for work. This may partly explain the difference in sex distribution between Sichuan and other countries. Over 80% of the victims were in the age group of 18–60-year-olds (Table 3), this age group of individuals were at greater risk of CO poisoning than other age groups. The similar age distribution was found in some countries (16). However, many other studies reported the eldest (4, 8, 17) were at the greatest risk of CO poisoning

In this study, most of the CO poisoning deaths were attributed to unintentional accidents, which was consistent with other studies (5, 21). About 65% of victims were died from showering gas accidents. According to investigations by the police, defective water heaters and/or improper installation were the primary risk factors in the most CO poisoning cases. Inadequate ventilation coupled with a longer shower in cold weather eventually caused most tragedies. This could largely explain the reason why unintentional accidents accounted for such large proportion.

CO poisoning mostly occurred indoor, hostel or rental house and the victims’ home were the most common places. Hostel or rental house especially the cheap rent ones usually had some features as follows: small space, old or inferior equipment, poor ventilation and so on. Migrant workers, needy families and many young couples preferred to stay in these places for the cheap rent. Another important reason was that people do not have to register the real names and identity card in these places. This was one of the most noticeable characteristics of CO poisoning in Sichuan.

Shanghai is a large city in eastern China, Wuhan locates in the center of China and Sichuan is in western China. There were significant regional differences in the manner of death and the source of CO. The percentage of unintentional accident was much higher than suicide in Shanghai and Sichuan. However, Wuhan was just the opposite. Based on the significant difference in the manner of death, the source of CO was divided into two groups: source of CO in suicide and in accident. In suicide cases, the source of CO in Wuhan and Sichuan were very similar. Coal or charcoal burning was the most common method, followed by gas leak. In Shanghai, nearly everyone committed suicide by burning coal or charcoal. The common methods of committing suicide vary from country to country. For example, the most common method of suicide with CO was by motor vehicle exhaust gases in Iran, Europe and United Stated (6, 17, 22). And charcoal burning has become one of the leading methods of suicide in Asia (23, 24). In accident cases, showering gas accident was the main source in Sichuan and Shanghai, but not in Wuhan. Gas leak was the first source, followed by Showering gas accident and coal or charcoal burning in Wuhan. Although what drives the differences is not clear, the findings of regional differences in CO poisoning will be useful, especially in unintentional CO poisoning precaution.

Like many other accidents and injuries, CO poisoning is considered preventable (25). Several pivotal factors associated with CO poisoning had been identified in this study. First, CO poisoning was seasonal with middle-aged people and young adults at the greatest risk. Second, rental house/hostel and home were the main sites of unintentional CO poisoning. Third, the focus for prevention in different regions should be variable according to the local features in CO poisoning. Prevention of indoor CO exposure in Sichuan can be accomplished as follows: 1) do not use inferior or improper installed water heater; 2) frequent inspection and routine maintenance of CO emitting equipment; 3) maintain indoor ventilation especially in cold weather; 4) install a CO detector; 5) spread preventive messages about potential source of CO by media especially to the high-risk groups.

## Conclusion

Non-fire related carbon monoxide poisoning remains a significant cause of death in mainland China. Basing on the analysis of CO poisoning deaths in Sichuan Province, several characteristics of CO poisoning have been identified. And the regional differences of CO poisoning have been proved by comparing the data of Sichuan, Shanghai, and Wuhan. These findings were beneficial to comprehensively understand the features of CO poisoning in mainland China and valuable in the prevention of CO poisoning. Raising the public awareness on the dangers of CO and popularizing the preventive messages about potential sources of CO exposure are pivotal in reducing incidence of poisoning. At the same time, sufficient precautions should be taken in time, and attention should be paid equally to both residences and workplaces.

## Ethical considerations

Ethical issues (Including plagiarism, informed consent, misconduct, data fabrication and/or falsification, double publication and/or submission, redundancy, etc.) have been completely observed by the authors.
